# Palynological Characteristics of Neogene Deposits from Bełchatów Lignite Mine (Central Poland)

**DOI:** 10.3390/plants14193034

**Published:** 2025-09-30

**Authors:** Thang Van Do, Ewa Durska

**Affiliations:** University of Warsaw, Faculty of Geology, Żwirki i Wigury 93, 02-089 Warsaw, Poland; edurska@uw.edu.pl

**Keywords:** Kleszczów Graben, Polish lowlands, Miocene, palaeoflora, palynology, pollen zone

## Abstract

The Bełchatów Lignite Mine (BLM) in central Poland, one of Europe’s largest Neogene lignite deposits, provides key insights into palaeofloral evolution. Located in the Kleszczów Graben, the BLM consists of four distinct lithological units: subcoal, coal, clayey-coal, and clayey-sandy units. The study presents a palynological investigation of 31 samples from all units, identifying 78 sporomorph taxa, including 10 plant spores, 15 gymnosperm pollen, and 53 angiosperm pollen taxa. Pollen grains from angiosperms and gymnosperms were consistently observed in all samples, while plant spores were scarce. The analysis reveals three distinct palynological zones, reflecting shifts in vegetation. The first zone is characterized by swamp, riparian, and mixed mesophilous forests, dominated by *Taxodium*/*Glyptostrobus*, *Ulmus*, *Carya*, *Engelhardia*, *Pterocarya*, and *Quercus*. In the second zone, slightly cooler climatic conditions led to the decline of *Taxodium*/*Glyptostrobus* and *Alnus*, indicating a deterioration of swamp forests. The third zone marks a subsequent recovery of these forests. Palaeoclimatic interpretations indicate three phases: a subtropical-humid climate during the Early Miocene, fluctuating humidity in the late Early Miocene, and a transition to a warm-temperate and humid climate in the Late Miocene.

## 1. Introduction

**Figure 1:**
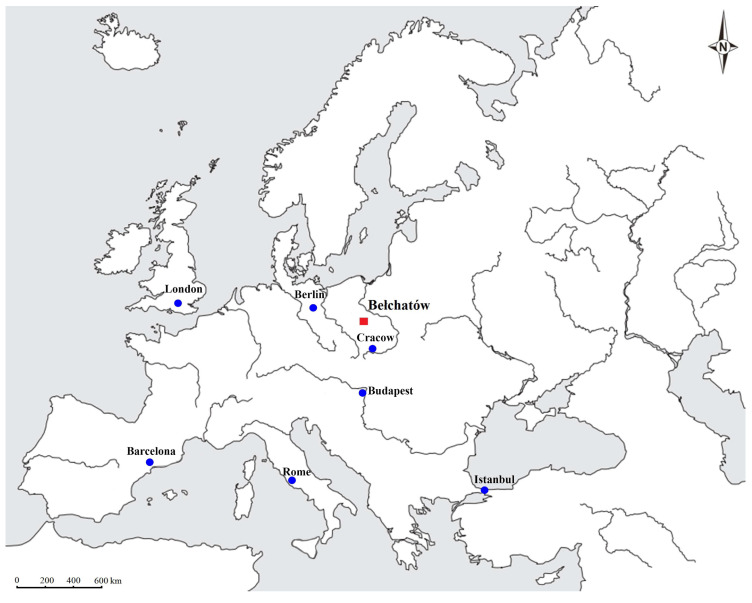
Location of the BLM, marked by a red color and the name “Bełchatów” (modified after Fostowicz-Frelik et al., 2012) [31].

The BLM (Figure 1) in central Poland is recognized as one of Europe’s largest Neogene lignite deposits, primarily exploited to satisfy the demand of the power plant industry. Its discovery dates back to the early 1960s. The BLM is situated within the tectonic depression known as the Kleszczów Graben, which has been filled with Neogene sediments containing lignite seams and plant macro- and microremains. The Neogene, particularly the Miocene epoch, witnessed significant global climatic shifts, including periods of warming like the Miocene Climatic Optimum, followed by a general cooling trend, which influenced regional ecosystems. Understanding the palaeoflora of the BLM provides a regional perspective on these broader global changes [1,2,3,4].

Significant research has been conducted on the fossilized plant remains [5,6,7,8,9,10,11,12,13,14,15,16,17], as well as other fossils such as algae [18], fungi [19,20], cladocera [21], insects [22], snails [23,24], freshwater fishes [25,26], and mammals [27,28,29,30,31] found within the Neogene deposits of the Kleszczów Graben.

**Figure 2:**
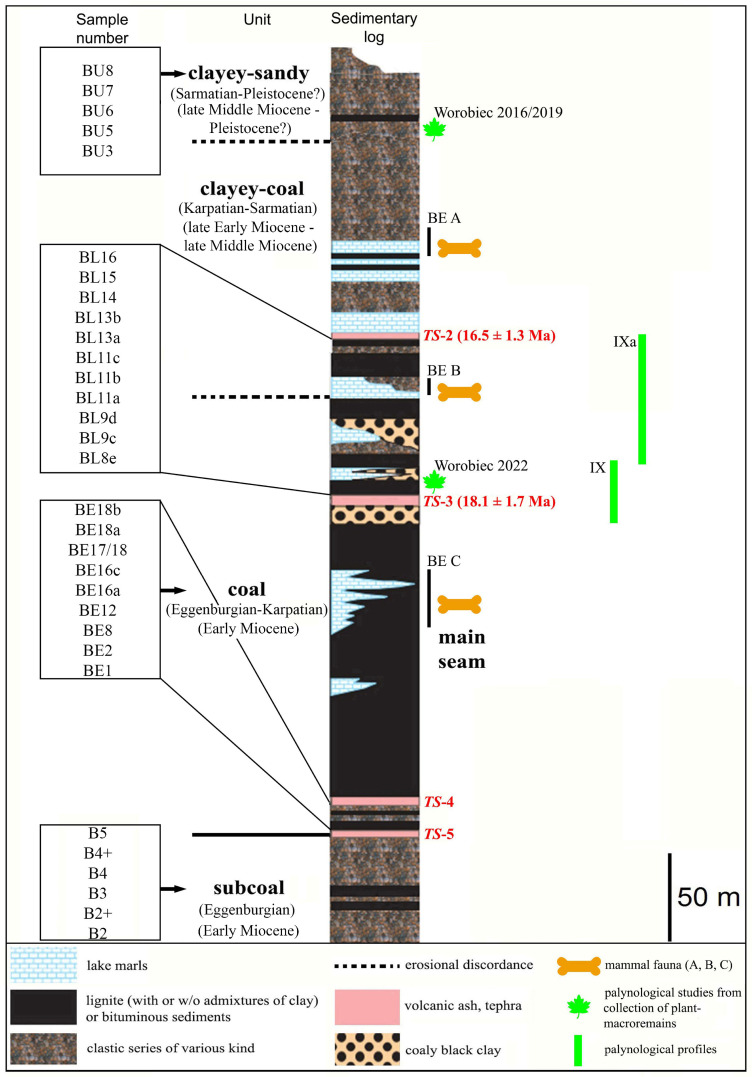
Generalized lithostratigraphic section of deposits of the BLM (modified after Fostowicz-Frelik et al., 2012) [31]. TS, tonsteins (track dating). B, BE, BL, BU: location of 31 samples used in the study. BE A, BE B, and BE C: mammal fauna horizons: Bełchatów A, Bełchatów B, and Bełchatów C by Kowalski (1993) [27]. Worobiec 2016/2019 and Worobiec 2022: locations of samples from palynological studies by Worobiec and Worobiec (2016) [33] and Worobiec and Worobiec (2019) [16] and Worobiec and Worobiec (2022) [34]. IX and IXa: palynological profiles of Stuchlik et al. (1990) [5].

According to Rzebik-Kowalska and Kowalski (2001) [28], fossil mammals are known in the BLM from three horizons (Figure 2). The uppermost horizon, Bełchatów A, contained several taxa of insectivores and at least 20 taxa of rodents representing 20 species. The intermediate horizon Bełchatów B, situated between two tuffic layers, predominantly contained small mammals. The lowermost fauna, in Bełchatów C, are situated below the lower tuffite layer. Among the large mammals were *Gomphotherium angustidens* and *Hypotherium soemmeringi*. Small mammals included insectivores and numerous rodents. The tooth of Megachiroptera collected belongs to the faunal assemblage.

The previous palynological analyses were not comprehensive. For example, Ziembińska-Tworzydło (1966) [32] conducted a study on a coal unit section without providing information about the number of samples analyzed and attributed the main seam within the coal unit to the Middle Miocene age. Stuchlik et al. (1990) [5] investigated 52 samples from profile IX/IXa (Figure 2) situated between the uppermost part of the coal unit and the lowermost part of the clayey-coal unit, which may indicate an Early Miocene (Karpatian) age. Worobiec and Worobiec (2016) [33], and Worobiec and Worobiec (2019) [16] (Figure 2), analyzed twelve samples from the clayey-sandy unit, which has been assigned an age ranging from the latest Middle Miocene to Late Miocene, while Worobiec and Worobiec (2022) [34] (Figure 2) analyzed eight samples and proposed an Early Miocene age for the formation of the coal unit.

This study shows investigation of Neogene deposits throughout the entire profile of BLM using palynological analyses with 31 samples. The well-preserved sporomorph association found in these samples exhibit relatively high taxonomical diversity, allowing for the reconstruction of plant communities. The results complement the existing data on palaeoflora and palaeovegetation from previous studies [5,16,32,33,34] and provide information on vegetation and palaeoclimate during the formation of the Bełchatów deposits.

### Geological Setting

The BLM is situated to the south of the town of Bełchatów in Central Poland (Figure 1). The Neogene sedimentary series of the Kleszczów Graben consists of four distinct lithological units, arranged from bottom to top: subcoal, coal, clayey-coal, and clayey-sandy unit (Figure 2) [35,36].

The main seam of the coal unit beneath the tonstein TS-3 (Figure 2) was dated by Burchart et al. (1988) [37] using the fission-track (FT) dating method. The age of the tonstein TS-3 is estimated to be 18.1 ± 1.7 Ma. Further down in the profile, the mammalian horizon Bełchatów C, belonging to the upper part of the main seam (Figure 2), likely represents the Upper Ottnangian (late Early Miocene) [30]. According to lithostratigraphic schemes of the Neogene in the Polish lowlands within a chronostratigraphic framework [38,39], the main coal seam belongs to the 3rd Ścinawa and perhaps the 2nd Lusatia coal seam group and was formed in the Early Miocene (Eggenburgian–Karpatian). The subcoal unit and lower-coal sediments were formed in the Early Miocene (Eggenburgian). Tonstein TS-2 (Figure 2) was dated at 16.5 ± 1.3 Ma [37]. Beneath it, an accumulation of mammalian remains (Bełchatów B) (Figure 2) was found, dated to be of Early Badenian (early Middle Miocene) age [27]. The coal unit was formed during the Eggenburgian–Karpatian (Early Miocene). The lowermost part of the clayey-coal unit is situated between the mammalian horizons Bełchatów B and the tonstein TS-2 (Figure 2). Based on lithostratigraphic schemes of the Neogene in the Polish lowlands within a chronostratigraphic framework [38,39], these deposits may have accumulated during the Karpatian (late Early Miocene). Further up in the profile, the mammalian horizon Bełchatów A, belonging to the upper part of the clayey-coal unit (Figure 2), probably indicates the Late Sarmatian–Early Pannonian (Late Middle to early Late Miocene) [30,31]. From the above discussion, it can be inferred that the clayey-coal unit was formed during the Karpatian–Sarmatian (late Early to early Late Miocene).

The lowermost part of the clayey-sandy unit, overlaying the mammalian horizon Bełchatów A (Figure 2), the studied palynological assemblages KRAM-P 218 [33] and KRAM-P 225 [16] (Figure 2) proposed these deposits originated during the late Middle Miocene (Sarmatian) and Late Miocene (early Pannonian). On the other hand, Wójcicki and Zastawniak (1998) [6] found new fossil species of *Trapa* in the clayey-sand layers examined in the clayey-sandy unit overlying the coal, suggesting a Pliocene age. Moreover, Worobiec and Lesiak (1998) [7] investigated the Stawek-1A site, which belongs to the clayey-sandy unit, and found a high percentage of herbs (57%) in the histogram, typically indicating a Late Pliocene age. Recently, Dumont et al. (2020) [21] discovered Bosminid fossils (cladoceran remains) in the clayey-sandy unit, previously known only from the Pleistocene. Based on the above discussion, it suggests that the clayey-sandy unit was formed during the Sarmatian to Pleistocene period (late Middle Miocene to Pleistocene).

As our study is based on palynological assemblages, a reliable and independent biostratigraphic or geochronological dating is particularly challenging to achieve with high precision. Therefore, our interpretations of the palaeovegetation and palaeoclimate are placed within this pre-existing chronological framework. We acknowledge the limitations of not undertaking an independent dating of our samples; however, this approach allows for a direct correlation of our palynological results with the well-documented geological history of the Bełchatów profile, providing a foundation for our palaeoenvironmental reconstructions.

## 2. Materials and Methods

A total of sixty-one sediment samples were collected from various sections across the entire BLM profile for comprehensive palynological investigations. These samples were macerated and analyzed; however, only thirty-one of them contained a sufficient number of palynomorphs for a detailed study. The results presented in this paper are based on these thirty-one positive samples (Figure 2) from the original collection of sixty-one. This archival material, currently stored at the Faculty of Geology, University of Warsaw, was originally collected from accessible parts of the excavation at approximately 1 m intervals or whenever there was a change in lithology. The samples cover (partially) four main lithological units: the subcoal unit and the lowermost part of the coal unit underlying the main coal seam, the uppermost part of the coal unit overlying the main coal seam, the lowermost part of the clayey-coal unit, and the clayey-sandy unit. Representative samples provide a foundation for reconstructing the palaeoenvironmental and palaeoclimatic evolution of the study area.

All samples underwent standard preparation methods for palynological analysis. Initially, the samples were crushed into smaller fragments, followed by the addition of 37.5% HCl to eliminate carbonates. Subsequently, a 70% HF solution was applied to dissolve silicate. The remaining residue was then sieved through a nylon mesh with a 10 μm aperture size. To separate undissolved mineral particles and organic matter, a ZnCl_2_ solution was added. Acetolysis, following the procedure described by Erdtman (1960) [40], was performed on the samples. Finally, slides were prepared using glycerine jelly as a mounting medium.

**Figure 3:**
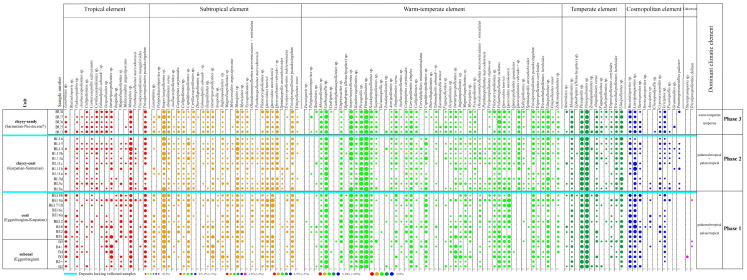
Simplified percentage pollen diagram of BLM arranged according to climatic requirements. Percentages grouped according to frequency: <0.5% (very rare), 0.5–1% (<1%) (rare), 1–2% (<2%) (low frequency), 2–5% (<5%) (moderate frequency), 5–10% (<10%) (high frequency), and ≥10% (dominant). Red circles: tropical taxa. Orange circles: subtropical taxa. Light green circles: warm-temperate taxa. Dark green circles: temperate taxa. Blue circles: cosmopolitan taxa. Purple circles: unknown taxa. Elements according to Stuchlik et al. (2001, 2002, 2009, 2014) [41,42,43,44].

**Figure 4:**
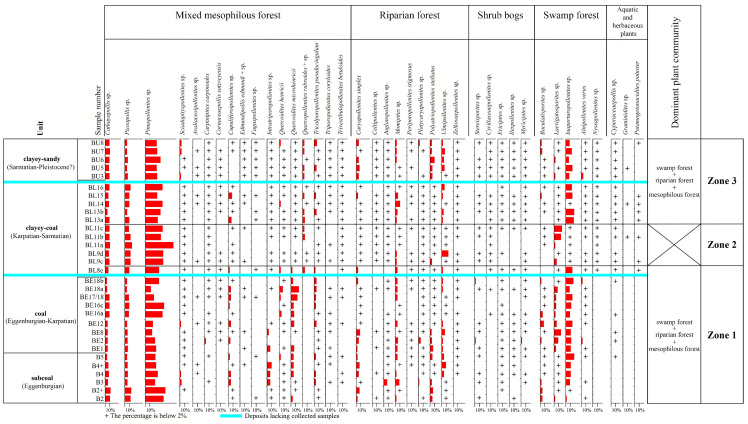
Simplified percentage pollen diagram of BLM (incl. plant community requirements).

Palynological analysis was performed using the light microscopes Olympus CX21 and Nikon Eclipse E600 POL at magnifications of 400×, 500×, and 1000× at the Faculty of Geology, University of Warsaw. A minimum of 300 sporomorphs were counted in each sample during the palynological analysis to enable the establishment of pollen diagrams (Figure 3 and Figure 4). These pollen diagrams were generated using the Xact and Corel (CorelDRAW X7) programs, with statistical analyses conducted using the Microsoft Excel 2007 program.

## 3. Results

**Figure 5:**
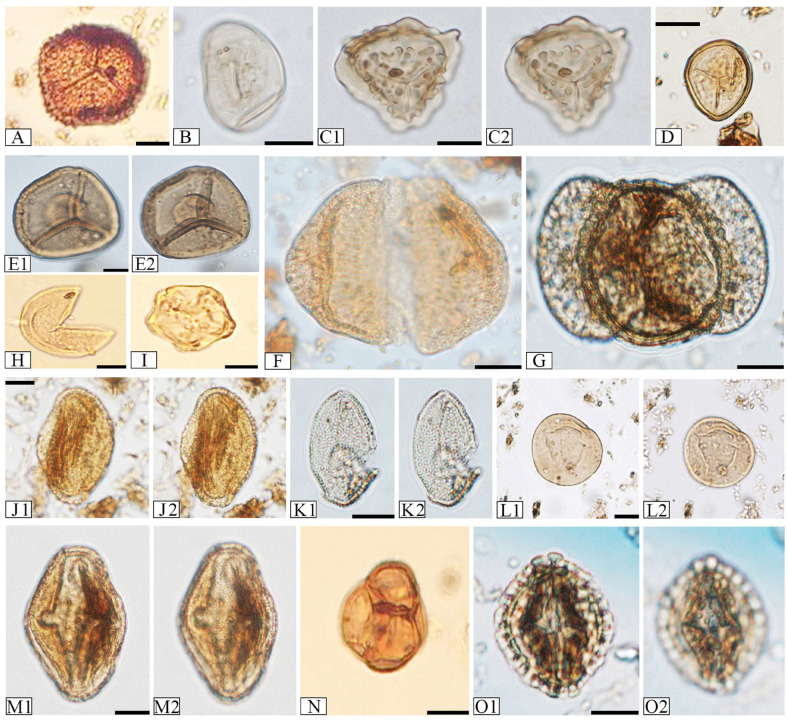
Microphotographs of identified spores and pollen grains. (**A**)—*Baculatisporites* sp. (*Osmunda*), magnification ×500. (**B**)—*Laevigatosporites* sp. (Polypodiaceae), magnification ×1000. (**C1**,**C2**)—*Polypodiaceoisporites* sp. (*Pteris*), same specimen, various foci, magnification ×1000. (**D**)—*Stereisporites* sp. (*Sphagnum*), magnification ×1000. (**E1**,**E2**)—*Leiotriletes* sp. (Lygodiaceae), same specimen, various foci, magnification ×500. (**F**)—*Cathayapollis* sp. (*Cathaya*), magnification ×1000. (**G**)—*Pinuspollenites* sp. (*Pinus*), magnification ×1000. (**H**)—*Inaperturopollenites* sp. (*Taxodium*/*Glyptostrobus*), magnification ×500. (**I**)—*Alnipollenites verus* (*Alnus*), magnification ×500. (**J1**,**J2**)—*Araliaceoipollenites* sp. (Araliaceae), same specimen, various foci, magnification ×1000. (**K1**,**K2)**—*Arecipites* sp. (Amaryllidaceae, Araceae, Arecaceae), same specimen, various foci, magnification ×1000. (**L1**,**L2**)—*Caryapollenites simplex* (*Carya*), same specimen, various foci, magnification ×500. (**M1**,**M2**)—*Edmundipollis edmundi* (Araliaceae), same specimen, various foci, magnification ×1000. (**N**)—*Ericipites* sp. (Ericaceae), magnification ×500. (**O1**,**O2**)—*Ilexpollenites iliacus* (*Ilex*), same specimen, various foci, magnification ×1000. Scale bars are 10 µm. Botanical affinity in brackets.

**Figure 6:**
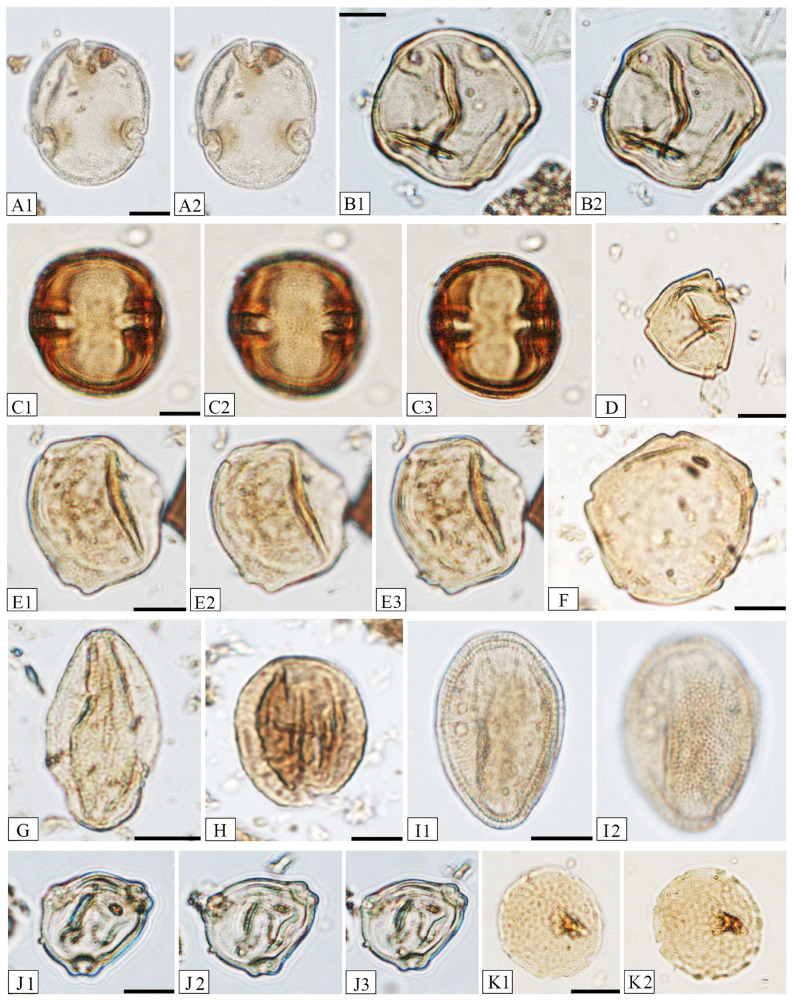
Microphotographs of identified pollen grains. (**A1**,**A2**)—*Intratriporopollenites* sp. (Brownlowioideae), same specimen, various foci, magnification ×1000. (**B1**,**B2**)—*Juglanspollenites* sp. (*Juglans*), same specimen, various foci, magnification ×1000. (**C1**–**C3**)—*Meliaceoidites angustiporatus* (Meliaceae), same specimen, various foci, magnification ×1000. (**D**)—*Momipites* sp. (*Engelhardia*), magnification ×500. (**E1**–**E3**)—*Myricipites* sp. (*Myrica*), same specimen, various foci, magnification ×1000. (**F**)—*Polyatriopollenites stellatus* (*Pterocarya*), magnification ×1000. (**G**)—*Quercoidites henricii* (*Quercus*), magnification ×1000. (**H**)—*Quercopollenites rubroides* (*Quercus*), magnification ×500. (**I1**,**I2**)—*Tricolporopollenites mangiferoides* (*Magnifera*), same specimen, various foci, magnification ×1000. (**J1**–**J3**)—*Trivestibulopollenites betuloides* (*Betula*), same specimen, various foci, magnification ×1000. (**K1**,**K2**)—*Ulmipollenites* sp. (*Ulmus*), same specimen, various foci, magnification ×1000. Scale bars are 10 µm. Botanical affinity in brackets.

The names of the nearest living relatives of the fossil plant taxa (Table S1) and their climatic requirements (Figure 3), based on Stuchlik et al. (2001, 2002, 2009, 2014) [41,42,43,44] and Słodkowska and Ziembińska-Tworzydło (2017) [45], are provided. Representative images of selected palynomorphs can be found in Figure 5 and Figure 6.

A total of 78 sporomorph taxa were identified, comprising 10 taxa of plant spores, 15 taxa of gymnosperm pollen, and 53 taxa of angiosperm pollen (Tables S1 and S2). Pollen grains from angiosperms and gymnosperms were consistently observed in all samples, while plant spores were scarce (Table S2). However, in samples including BE2, BE8, BE12, BE18a, BL11b, and BL11c (Table S2), plant spores were frequently observed, ranging from 10.3% to 17.8%, with dominant taxa including *Laevigatosporites* sp. (5.6–16.2%), *Baculatisporites* sp. (0.3–6.9%), *Stereisporites* sp. (0.3–2.0%), and *Leiotriletes* sp. (not exceeding 2.0%). In most samples (Table S2), pollen grains of gymnosperms were most abundant containing *Pinuspollenites* sp. (12.3–60.3%), *Inaperturopollenites* sp. (0.3–19.9%), *Piceapollis* sp. (3.2–13.9%), *Cathayapollis* sp. (3.6–12.3%), *Cupressacites* sp. (up to 5.3%), *Sciadopityspollenites* sp. (up to 2.9%), *Sequoiapollenites* sp. (0.3–2.3%), *Abiespollenites* sp. (0.3–2.2%), *Keteleeriapollenites* sp. (0.3–1.3%), *Zonalapollenites* sp. (not exceeding 1.0%), *Podocarpidites* sp. (not exceeding 0.7%), *Cedripites* sp. (rarely observed, not exceeding 0.3%), *Cunninghamiaepollenites* sp. (not exceeding 0.3%), *Ephedripites* (*Distachyapites*) sp. (rarely observed, not exceeding 0.3%), and *Taiwaniapollis* sp. (rarely observed, not exceeding 0.3%). Angiosperms exhibit greater diversity including *Quercoidites microhenricii* (up to 17.8%), *Ulmipollenites* sp. (0.3–14.9%), *Polyatriopollenites stellatus* (0.6–11.1%), *Momipites* sp. (0.3–10.0%), *Intratriporopollenites* sp. (up to 9.4%), *Caryapollenites simplex* (0.3–8.3%), *Cupuliferoipollenites* sp. (up to 6.6%), *Juglanspollenites* sp. (up to 6.6%), *Quercoidites henricii* (up to 6.2%), *Tricolporopollenites pseudocingulum* (up to 4.9%), *Quercopollenites rubroides* and *Quercopollenites* sp. (up to 4.0%), *Platycaryapollenites* sp. (up to 3.7%), *Alnipollenites verus* (up to 3.0%), *Magnoliaepollenites* sp. (up to 2.2%), *Carpinipites carpinoides* (up to 2.0%), *Myricipites* sp. (up to 2.0%), *Ilexpollenites* sp. (up to 1.9%), *Cyperaceaepollis* sp. (up to 1.7%), *Oleoidearumpollenites microreticulatus* and *Oleoidearumpollenites reticulatus* (up to 1.7%), *Trivestibulopollenites betuloides* (up to 1.7%), *Ericipites* sp. (up to 1.6%), *Zelkovaepollenites* sp. (up to 1.5%), *Cyrillaceaepollenites* sp. (up to 1.3%), *Faguspollenites* sp. (up to 1.3%), *Aceripollenites* sp. (up to 1.0%), *Celtipollenites* sp. (not exceeding 1.0%), *Cercidiphyllites minimireticulatus* (up to 1.0%), *Periporopollenites stigmosus* (up to 1.0%), *Arecipites* sp. (not exceeding 0.9%), *Fraxinipollis* sp. (not exceeding 0.9%), *Cornaceaepollis satzveyensis* (not exceeding 0.7%), *Edmundipollis edmundi* and *Edmundipollis* sp. (not exceeding 0.7%), *Eucommiapollis minor* (0.7%), *Graminidites* sp. (0.7%), *Nyssapollenites* sp. (not exceeding 0.7%), *Tricolporopollenites indeterminatus* (up to 0.7%), *Triporopollenites coryloides* (not exceeding 0.7%), *Iteapollis* sp. (not exceeding 0.6%), *Parthenopollenites marcodurensis* (not exceeding 0.6%), *Potamogetonacidites paluster* (not exceeding 0.6%), *Salixipollenites* sp. (up to 0.6%), *Spinulaepollis arceuthobioides* (not exceeding 0.6%), *Vitispollenites tener* (up to 0.6%), *Araliaceoipollenites* sp. (up to 0.3%), *Chenopodipollis* sp. (not exceeding 0.3%), *Diervillapollenites* sp. (not exceeding 0.3%), *Meliaceoidites angustiporatus* (up to 0.3%), *Quercopollenites granulatus* (not exceeding 0.3%), *Tricolporopollenites dolium* (not exceeding 0.3%), and *Tricolporopollenites mangiferoides* (not exceeding 0.3%). Nevertheless, in sample BE18a (Table S2), angiospermous pollen grains are more abundant than gymnospermous pollen grains reaching 57.9% vs. 31.8%.

## 4. Discussion

### 4.1. Palaeovegetation and Palaeoenvironment

Based on the taxonomic composition of the samples and frequencies of sporomorph taxa, three zones (Figure 4) were distinguished.Zone 1 (B2-BL8e samples) is characterized by the dominance of swamp, riparian, and mixed mesophilous forests. Riparian forests, particularly in the lower part of Zone 1 (B2–BE12 samples), were primarily composed of *Ulmus*, *Carya*, *Engelhardia*, *Pterocarya*, *Juglans*, and *Platycarya*, along with *Zelkova*, *Liquidambar*, and *Celtis*.

Swamp forests were characterized by the dominance of *Taxodium*/*Glyptostrobus* and *Alnus*. Shrub bogs included *Myrica*, *Sphagnum*, *Ilex*, and members of the Ericaceae family, accompanied by Cyrillaceae and Clethraceae. The undergrowth likely featured ferns from Polypodiaceae, Davalliaceae, and *Osmunda*.

The presence of *Quercus* (represented by fossil-species *Quercoidites henricii* and *Quercoidites microhenricii*), *Cupuliferoipollenites* sp. (Fagaceae: *Castanea*, *Castanopsis*, *Lithocarpus*), Brownlowioideae, Tilioideae, plants producing pollen of the fossil-species *Tricolporopollenites pseudocingulum* (Fagaceae), *Carpinus*, along with Araliaceae, Mastixiaceae, Cornaceae, *Fagus*, *Corylus*, and *Betula*, suggests a well-developed mixed mesophilous forest. Pollen grains of *Pinus*, *Abies*, *Tsuga*, *Cathaya*, and *Picea* may have originated from plant communities on elevated terrain distant from the deposition area [33,46,47,48], or, alternatively, from trees within mixed mesophytic or even wet forests, potentially as an admixture with other species [33,46,49]. Some species of *Pinus* and *Sciadopitys* might have been present in the vicinity of the water body [33,50,51].

The occurrence of *Potamogeton* pollen in sample BL8e indicates a freshwater body, likely bordered by swamp vegetation (*Taxodium*/*Glyptostrobus*, *Alnus*, and *Nyssa*), herbs (including members of the Cyperaceae family), riparian forests (*Engelhardia*, *Carya*, and *Ulmus*, accompanied by *Pterocarya*, *Platycarya*, *Zelkova*, and *Liquidambar*), and shrub bogs (*Myrica*, *Ilex*, Ericaceae, Cyrillaceae, Clethraceae, Polypodiaceae, Davalliaceae, and other ferns). The mixed mesophilous forests included *Quercus*, *Cupuliferoipollenites* sp. (Fagaceae: *Castanea*, *Castanopsis*, *Lithocarpus*), plants producing pollen of the fossil-species, *Tricolporopollenites pseudocingulum*, and conifers, along with Brownlowioideae, Tilioideae, *Carpinus*, Mastixiaceae, *Fagus*, and *Betula*. The palynoflora in sample BL8e is highly similar to the palynological assemblage reported in the KRAM-P 214 collection of plant macroremains [34] (Figure 2), both in taxonomic composition and relative abundance. In particular, the proportions of plant spores, gymnosperms, and angiosperms in sample BL8e are 0.7%, 66.6%, and 32.8%, while those in the KRAM-P 214 sample are 0.9%, 67.9%, and 31.2%, respectively. These results demonstrate a remarkable similarity between the two studies. Moreover, the presence of *Potamogeton*, which is indicative of a freshwater environment, is common to both assemblages.

The dominance in Zone 1 of diverse mixed mesophilous forests including *Quercus*, *Carpinus*, *Corylus*, and various conifers, accompanied by swamp and riparian communities, characterized primarily by the presence of *Taxodium*/*Glyptostrobus*, *Ulmus*, and *Carya*, is consistent with regional palaeovegetation records across other Central European sites, such as the Lusatian lignite deposits in Germany [47,50,51] and Polish lignite deposits from western Poland [48,52].

Zone 2 (BL9c-BL11c samples) shows a deterioration in favorable conditions for swamp forests, evidenced by a decline in the frequencies of *Taxodium*/*Glyptostrobus* and *Alnus*. The presence of *Potamogeton* pollen indicates the existence of a water body. *Potamogeton* occurs inconsistently throughout Zone 2, but its frequent appearance suggests a persistent water source. However, overall vegetation diversity decreased in relation to the previous zone. Sample BL11a exhibits a pronounced dominance of conifers (89.7%), particularly *Cathaya* (11.3%), *Picea* (13.9%), and *Pinus* (60.3%), suggesting changes of climatic conditions that contributed to reduced plant diversity.

Despite these shifts, swamp vegetation continued to border the water body, and was dominated by *Taxodium*/*Glyptostrobus*, *Alnus*, *Nyssa*, and herbaceous plants (Cyperaceae and Poaceae). Riparian forests included *Ulmus*, *Engelhardia*, and *Pterocarya*, alongside *Carya*, *Celtis*, *Juglans*, *Liquidambar*, *Platycarya*, and *Zelkova*. Shrub bogs comprised *Myrica*, *Ilex*, and members of the Ericaceae family, Cyrillaceae, Clethraceae, Polypodiaceae, Davalliaceae, and other ferns. Mixed mesophilous forests showed a reduced representation of *Quercus* (plants producing pollen of the fossil-species *Quercoidites henricii*, *Quercoidites microhenricii*, *Quercopollenites rubroides*, and *Quercopollenites* sp.), *Betula*, *Carpinus*, conifers, and *Cupuliferoipollenites* sp. (Fagaceae: *Castanea*, *Castanopsis*, *Lithocarpus*), plants producing pollen of the fossil-species *Tricolporopollenites pseudocingulum*, as well as Brownlowioideae, Tilioideae, Mastixiaceae, Cornaceae, and Araliaceae.

Zone 3 (BL13a-BU8 samples) is characterized by environments favorable again for swamp forests, with a significant share of *Taxodium*/*Glyptostrobus*, *Alnus*, and *Nyssa* trees. Members of the families Ericaceae, Cyrillaceae, and Clethraceae, *Ilex*, and *Sphagnum*, as well as *Myrica*, could have formed bush swamps. The undergrowth likely included *Osmunda*, Polypodiaceae, Davalliaceae, and other ferns. The pollen assemblage points to the dominance of wetland vegetation, primarily riparian, during sedimentation. Vegetation becomes more diversified than in the previous zone.

The presence of *Potamogeton* pollen in multiple samples throughout Zone 3, indicates the existence of a freshwater body surrounded by swamp vegetation comprising herbs (including members of the families Cyperaceae and Poaceae) and riparian forests consisting of *Ulmus*, *Carya*, *Engelhardia*, *Pterocarya*, and *Platycarya*, accompanied by *Zelkova*, *Celtis*, *Juglans*, and *Liquidambar*.

Mixed mesophilous forests exhibited the higher species richness compared to Zone 2, including *Quercus*, plants producing pollen of the fossil-species *Tricolporopollenites pseudocingulum*, *Cupuliferoipollenites* sp. (Fagaceae: *Castanea*, *Castanopsis*, *Lithocarpus*), Brownlowioideae, Tilioideae, *Carpinus*, *Betula*, *Corylus*, and conifers, along with Araliaceae, Mastixiaceae, Cornaceae, and *Fagus*. Although the exact spatial relationship between the sample BU3 and samples from the KRAM-P 218 [33] and KRAM-P 225 [16] remains uncertain, a comparison of their palynological compositions provides valuable insights into local palaeovegetational variability. In sample BU3, plant spores account for 6.6%, compared to 3.3% in the KRAM-P 218 and KRAM-P 225 samples. Gymnosperms represent 57.6% in sample BU3, whereas they comprise 47.9% in the reference results. Angiosperms constitute 35.8% in sample BU3, in contrast to 48.8% in the comparative samples.

### 4.2. Palaeoclimate

Based on the frequency of sporomorph taxa classified into climatic elements, following Stuchlik et al. (2001, 2002, 2009, 2014) [41,42,43,44], three palaeoclimatic phases were identified (Figure 3).

Phase 1 (B2-BE18b samples) is characterized by the high frequency of palaeotropical and palaeosubtropical taxa, mainly including Lygodiaceae, *Podocarpus*, *Ilex*, Brownlowioideae, Tilioideae, *Engelhardia*, *Tricolporopollenites pseudocingulum*, *Taxodium*/*Glyptostrobus*, *Alnus*, *Carpinus*, *Cupuliferoipollenites* sp. (Fagaceae: *Castanea*, *Castanopsis*, *Lithocarpus*), Cornaceae, Mastixiaceae, Araliaceae, *Juglans*, *Magnolia*, *Myrica*, Oleaceae, *Platycarya*, and *Quercus*. These taxa are accompanied by *Schizaea*, Araliaceae, *Celtis*, Cyrillaceae, Iteaceae, Meliaceae, *Parthenopollenites marcodurensis* (Vitaceae), *Mangifera*, *Pteris*, *Arceuthobium*, Hamamelidaceae, and *Vitispollenites tener* (*Vitis*).

This phase is characterized by the highest representation of tropical elements in the entire BLM with a total of 48 taxa, including 18 palaeotropical and 30 palaeosubtropical. The presence of cosmopolitan taxa (*Osmunda*, *Sphagnum*, Polypodiaceae, Davalliaceae, Ericaceae, Cyperaceae), along with pollen from Arecaceae or possibly other families such as Amaryllidaceae, Araceae, and Butomaceae, and riparian and swamp forests indicators suggests a humid climate. These data indicate that the vegetation during the Early Miocene (Eggenburgian–Karpatian), when the lowermost part of the BLM sediments was deposited, developed under a subtropical and humid climate, and appears to be the warmest period recorded in the BLM.

Phase 1 probably reflects Miocene Climate Optimum, that occurred between 16.9 and 14.7 Ma in Central Europe [4]. The presence of Brownlowioideae, *Engelhardia*, Mastixiaceae, Araliaceae, *Ilex*, and Lygodiaceae provides a more direct link to the warm-temperate to subtropical conditions associated with the Miocene Climate Optimum.

During Phase 2 (BL8e-BL16 samples) the palaeotropical and palaeosubtropical taxa are still frequent, including *Podocarpus*, *Celtis*, Mastixiaceae, Cyrillaceae, *Ilex*, Brownlowioideae, Tilioideae, *Engelhardia*, *Tricolporopollenites pseudocingulum*, *Taxodium*/*Glyptostrobus*, *Alnus*, *Carpinus*, Fagaceae (*Castanea*, *Castanopsis*, *Lithocarpus*), *Juglans*, *Magnolia*, *Myrica*, *Nyssa*, Oleaceae, *Platycarya*, and *Quercus*. In addition, other taxa, including Lygodiaceae, Araliaceae, Cornaceae, Iteaceae, Meliaceae, *Pteris*, *Parthenopollenites marcodurensis* (Vitaceae), *Arceuthobium*, Hamamelidaceae, and *Vitispollenites tener* (*Vitis*), are also present.

Palaeosubtropical taxa (32 species) remain dominant among tropical elements, while palaeotropical taxa exhibit a slight decline, decreasing to 16. However, this reduction is minor, and a more reliable indicator of climatic trends seems to be a shift in a relative frequency of warm-temperate and temperate taxa (Figure 3). Their presence shows a more regular pattern than during Phase 1, both in terms of their appearance in the samples and their frequency, indicating a tendency toward a gentle cooling trend compared to Phase 1.

Humidity conditions within Phase 2 appear to be varied. In the lower part of Phase 2 (BL8e-BL11c samples), apart from sample BL8e, which exhibits a high frequency of *Taxodium*/*Glyptostrobus*, its occurrence remains low, suggesting that swamp forest conditions were temporarily less favorable. This decline could indicate drier local conditions affecting wetland vegetation. However, in the upper part of Phase 2 (BL13a-BL16 samples), *Taxodium*/*Glyptostrobus* reappears frequently, indicating the return of wetland conditions.

Despite these fluctuations, the presence of aquatic taxa, particularly *Potamogeton*, alongside the frequent occurrence of freshwater dinoflagellates and *Pediastrum* algae, suggests that freshwater availability remained relatively stable throughout this phase. Additionally, the frequent occurrence of cosmopolitan taxa (Polypodiaceae, Davalliaceae, *Osmunda*, *Sphagnum*, Ericaceae, Cyperaceae, *Potamogeton*, *Fraxinus*, Arecaceae, and Poaceae) and the expansion of water-adapted, riparian, and swamp forest plants indicate persistently high humidity. However, rather than a continuous increase in humidity compared to Phase 1, these data suggest episodic variations in moisture levels within a generally humid climate.

On the other hand, a significant shift is observed at sample BL11a, where a marked increase in warm-temperate and temperate elements (*Cathaya*, *Picea*, *Pinus*, and *Sciadopitys*) likely indicates a trend toward slightly cooler conditions within an overall subtropical climatic condition. The dominance of warm-temperate and temperate taxa (*Cathaya*, *Sciadopitys*, *Acer*, *Carya*, *Fagus*, *Pterocarya*, *Corylus*, *Betula*, *Ulmus*, and *Zelkova*) in sample BL16 further suggests a gentle climatic shift toward Phase 3.

Palynological evidence from Worobiec and Worobiec (2022) [34], based on samples probably collected from the horizon corresponding to the sample BL8e level (Figure 2), indicates a subtropical and humid climate. This interval likely falls at the boundary between Phases 1 and 2 in this study. Accordingly, their interpretation represents an earlier stage that precedes the emergence of cooler climatic trends observed in the upper part of the section. Fossil evidence supports the presence of a warm and humid environment during that time, including the fruit-eating bat (Pteropodidae, Megachiroptera) from the Lower Miocene deposits [28] in mammalian horizon Bełchatów C (Figure 2), as well as remains of the thermophilic freshwater fish *Esox sibiricus* [26] found in the same horizon.

From the above data, it can be concluded that during Phase 2, which occurred from the late Early Miocene (Karpatian) to the early Middle Miocene (early Badenian) [27,37,38,39], vegetation developed in a subtropical and humid climate. However, the observed variations in *Taxodium*/*Glyptostrobus* and other wetland indicators suggest that moisture availability was not uniform and likely fluctuated within Phase 2 rather than showing a consistent increase relative to Phase 1.

Phase 3 (BU3-BU8 samples) represents a distinct climatic transition, as evidenced by the increased continuity and dominance of warm-temperate and temperate elements across all samples. Although the total number of warm-temperate taxa is slightly lower (44 species) compared to Phase 1 (49 species) and Phase 2 (50 species), their frequency and regularity of occurrence are notably higher in Phase 3. Taxa such as *Abies*, *Cathaya*, *Picea*, *Pinus*, *Sciadopitys*, *Sequoia*, *Acer*, *Carpinus*, *Fagus*, Tilioideae, *Pterocarya*, *Ulmus*, and *Zelkova* are consistently present throughout this phase, indicating a stable representation of warm-temperate climatic conditions.

Furthermore, temperate elements including *Betula*, *Corylus*, *Fagus*, *Ulmus*, and *Zelkova* exhibit a high degree of continuity, maintaining a steady presence in mostly all samples. In contrast, their occurrence in Phases 1 and 2 is more sporadic and irregular. This supports the interpretation of a gradual shift toward more temperate environmental conditions during Phase 3.

At the same time, the relative abundance and diversity of tropical and subtropical taxa slightly decline. The number of palaeotropical species decreases to 13, and palaeosubtropical species drop to 27. These taxa also appear less frequently and with reduced dominance compared to earlier phases. This decline, combined with the stable and widespread presence of temperate and warm-temperate taxa, suggests a transition toward a cooler and more seasonally variable climate. It probably reflects the Middle to Late Miocene cooling trend observed in Europe [53,54,55].

Cosmopolitan taxa, including *Osmunda*, Polypodiaceae, Davalliaceae, *Sphagnum*, Ericaceae, Cyperaceae, *Potamogeton*, *Fraxinus*, Poaceae, and Chenopodiaceae, continue to be well represented in Phase 3, indicating the persistence of moisture-dependent vegetation. The presence of riparian and swamp forest elements suggests that the climate remained humid.

Based on these data, it can be concluded that during Phase 3 (late Middle Miocene-Late Miocene), vegetation at BLM developed under a warm-temperate and consistently humid climate. This finding corresponds with previous revisions by Worobiec and Worobiec (2016) [33] and Worobiec and Worobiec (2019) [16], which characterized the Late Miocene climate at BLM as warm-temperate and moderately wet.

The persistence of high humidity at BLM contrasts with some research suggesting increasing aridification in certain inland areas of Central Europe during the Late Miocene [4]. This divergence highlights the importance of local controls which may have included factors such as the presence of large fluvial and lacustrine systems associated with the Kleszczów Graben, potentially providing specific conditions including stable groundwater levels, what allowed Bełchatów region to function as a humid refugium.

## 5. Conclusions

The palynological analysis of 31 samples from the BLM has provided valuable insights into the palaeovegetation and palaeoclimate during the Miocene. A total of 78 sporomorph taxa were identified, including 10 plant spore taxa, 15 gymnosperm pollen taxa, and 53 angiosperm pollen taxa. Based on their composition and frequency variations, three palaeovegetational zones and three distinct palaeoclimatic phases were distinguished.

Zone 1 was dominated by swamp, riparian, and mixed mesophilous forests, with *Taxodium*/*Glyptostrobus*, and *Alnus* as key wetland taxa. Riparian forests composed of *Ulmus*, *Carya*, *Engelhardia*, *Pterocarya*, and *Juglans* were well developed. A diverse mixed mesophilous forest included *Quercus* (represented by fossil-species *Quercoidites henricii* and *Quercoidites microhenricii*), *Cupuliferoipollenites* sp. (Fagaceae: *Castanea*, *Castanopsis*, *Lithocarpus*), Brownlowioideae, Tilioideae, plants producing pollen of the fossil-species *Tricolporopollenites pseudocingulum*, *Carpinus*, and conifers. Zone 2 shows a decline in swamp forest taxa and an increase in conifers such as *Pinus*, *Picea*, and *Cathaya*, indicating slightly cooler climatic conditions. Gymnosperms peaked at 89.7% in sample BL11a, marking a major ecological shift. Zone 3 reveals a recovery of swamp forests and increased diversity in riparian and mesophilous forests.

The palaeoclimatic reconstruction indicates a transition from a subtropical-humid climate in the Early Miocene (Phase 1) to a period of fluctuating humidity in the late Early and Middle Miocene (Phase 2), followed by a shift to a warm-temperate and humid climate in the late Middle and Late Miocene (Phase 3). Phase 1 is characterized by the highest representation of palaeotropical and palaeosubtropical taxa, pointing the warmest period recorded at BLM and reflecting the Miocene Climatic Optimum. In Phase 2 a decline in vegetation diversity and a coniferous dominance reflect change to slightly drier and cooler climate. In Phase 3 the persistent and dominant presence of warm-temperate and temperate taxa, along with the marked decline in palaeotropical and palaeosubtropical elements, suggest a transition toward a cooler and more seasonally variable environment.

The Bełchatów profile records a gradual climatic transition from Early Miocene subtropical-humid conditions to Late Miocene warm-temperate conditions. This aligns with broader regional trends.

## Data Availability

The datasets analyzed during the current study are available from the corresponding author upon reasonable request.

## Supplementary Materials

The following supporting information can be downloaded at https://www.mdpi.com/article/10.3390/plants14193034/s1, Table S1: Nearest living relatives of fossil taxa (based on Stuchlik et al., 2001, 2002, 2009, 2014) [41,42,43,44]; Table S2: Results of palynological analysis (their percentages) from BLM.

## Abbreviation

The following abbreviation is used in this manuscript:

BLM	Bełchatów Lignite Mine
